# Machine-learning based quantification of lung shunt fraction from 99mTc-MAA SPECT/CT for selective internal radiation therapy of liver tumors using TriDFusion (3DF)

**DOI:** 10.1186/s40658-025-00732-9

**Published:** 2025-03-11

**Authors:** Daniel Lafontaine, Finn Augensen, Adam Kesner, Raoul Vincent, Assen Kirov, Simone Krebs, Heiko Schöder, John L. Humm

**Affiliations:** 1 Departments of Medical Physics, Memorial Sloan Kettering Cancer Center, 1250 First Avenue, New York, NY 10065 USA; 2 Departments of Radiology, Memorial Sloan Kettering Cancer Center, 1250 First Avenue, New York, NY 10065 USA

**Keywords:** Lung shunt fraction, Selective internal radiation therapy, 99mTc- macroaggregated albumin, TriDFusion (3DF), Machine learning

## Abstract

**Background:**

Prior to selective internal radiotherapy of liver tumors, a determination of the lung shunt fraction (LSF) is performed using 99mTc- macroaggregated albumin (99mTc-MAA) injected into the hepatic artery. Most commonly planar but sometimes SPECT/CT images are acquired upon which regions of interests are drawn manually to define the liver and the lung. The LSF is then calculated by taking the count ratios between these two organs. An accurate estimation of the LSF is necessary to avoid an excessive pulmonary irradiation dose.

**Methods:**

In this study, we propose a computational, semi-automatic approach for LSF calculation from SPECT/CT scans, based on machine learning 3D segmentation, implemented within TriDFusion (3DF). We retrospectively compared this approach with the LSF calculated using the standard planar approach on 150 patients. Using CT images (from the SPECT/CT) as a blueprint, the TotalSegmentor machine learning algorithm automatically computes masks for the liver and lungs. Then, the SPECT attenuation-corrected images are fused with the CT and, based on the CT segmentation mask, TriDFusion (3DF) generates volume-of- interest (VOI) regions on the SPECT images. The liver and lung VOIs are further augmented to compensate for breathing motion. Finally, the LSF is calculated using the number of counts in the respective VOIs. Measurements using an anthropomorphic 3D-printed phantom with variable 99mTc activity concentrations for the liver and lungs were performed to validate the accuracy of the algorithm.

**Results:**

On average, LSF determined from 2D planar images were between 21 and 70% higher than those determined from SPECT/CT data. Semi-automated determination of the LSF using TriDFusion (3DF) analysis of SPECT-CT acquisitions was within 4–12% of the phantom-determined ratio measurements (ground truth).

**Conclusions:**

The utilization of TriDFusion (3DF) AI 3D Lung Shunt is a precise method for quantifying lung shunt fraction (LSF) and is more accurate than planar 2D image-based estimates. By incorporating machine learning segmentation and compensating for breathing motion, the approach underscores the potential of artificial intelligence (AI)-driven techniques to revolutionize pulmonary imaging, providing clinicians with efficient and reliable tools for treatment planning and patient management.

## Background

Selective internal radiation therapy (SIRT) [1, 2], employing yttrium-90 (90Y) microspheres, represents a pivotal intervention in the management of liver malignancies. Essential to the safe and effective delivery of SIRT is the accurate evaluation of the liver-lung shunt fraction (LSF) [3–7], which serves as a critical determinant in mitigating the risk of radiation pneumonitis [8]. Presently, the assessment of LSF predominantly relies on planar imaging techniques, necessitating manual segmentation processes. However, these conventional methods are fraught with inherent limitations, characterized by their time-intensive nature and susceptibility to subjective interpretation, often contingent upon the expertise of the involved technologists. As such, there exists a pressing need for advanced imaging methodologies that offer enhanced accuracy, efficiency, and reproducibility in the assessment of LSF and optimize the workflow of SIRT planning.

## Methods

The semi-automated LSF software is an application built as an open-source, extensible medical viewing platform called TriDFusion (3DF) [9]. This software is designed specifically to address the unmet needs of nuclear medicine clinical imaging in research studies. TriDFusion (3DF) Image Viewer utilizes TotalSegmentor [10], an advanced open-source segmentation tool based on nnU-Net [11], to automate the segmentation of liver and lungs in CT scans. The process entails the following steps:

### Liver and lung segmentation

TriDFusion (3DF) pulls SPECT-CT data from either a single or multi-field SPECT-CT acquisition containing the liver and lung anatomical and functional information required for the LSF estimation. TotalSegmentor is employed to perform automatic segmentation of the liver and lungs based on the CT images. This advanced segmentation tool utilizes state-of-the-art algorithms to accurately delineate the boundaries of these organs. Based on the CT segmentation mask, TriDFusion (3DF) generates volume-of-interest (VOI) regions on the SPECT attenuation-corrected (AC) images. These VOIs define the source areas of interest within the SPECT data for further analysis.

### Accurate segmentation and augmentation technique

To correct for respiratory motion artifacts [12] and ensure that the intensity of the SPECT data is contained within the organ VOIs of the segmented CT, TriDFusion (3DF) applies a pixel augmentation strategy. The augmented liver ROIs are adjusted to avoid overlap with the lung ROIs. This ensures accurate separation of the lung and liver source regions, minimizing interference between the two. TriDFusion (3DF) provides the flexibility to allow users to fine-tune the parameters based on specific clinical requirements. It also provides a tool to correct for missing lung tissue if the top of the lung is cropped in the SPECT-CT acquisition.

To correct for respiratory motion artifacts [12] and ensure that the intensity of the SPECT data is contained within the organ VOIs of the segmented CT, TriDFusion (3DF) applies a pixel augmentation strategy. The augmented liver VOI are adjusted to avoid overlap with the lung VOI. This ensures accurate separation of the lung and liver source regions, minimizing interference between the two. TriDFusion (3DF) provides the flexibility to allow users to fine-tune the parameters based on specific clinical requirements. The machine learning segmentation process is highly efficient, taking approximately one minute to complete depending on the graphic card processing unit (GPU). Visual inspection of the results is generally rapid, and in most cases, no manual adjustment is required. In our cohort of 150 cases, only a small proportion (<6%) required further adjustments, with manual corrections typically taking less than two minutes per case. However, to account for potential segmentation inaccuracies, TriDFusion (3DF) includes advanced editing tools, such as a 2D brush, to facilitate necessary refinements of the ROIs. While human intervention is minimized, an evaluation of LSF accuracy before and after manual edits showed an average error difference of ~18% (range 5–30%), highlighting the importance of manual corrections for optimal LSF estimation.

### Generation of a report

TriDFusion (3DF) includes an automated report generation feature that provides a comprehensive summary of the LSF analysis. The report includes patient-specific information about the series, such as demographic details and relevant clinical data. Additionally, a 3D rendering of the series, showcasing the segmented lung shunt regions, is incorporated into the report. Any pixel augmentation or dimensional adjustment is recorded in the generated LSF report. Furthermore, the software provides the ability to perform manual inspection and adjustments of the Lung and Liver Regions of Interest (ROIs). The LSF is then recalculated should there be any edited contours.

### Validation, 3D-printed phantom creation, image acquisition, and reconstruction

**Fig. 1:**
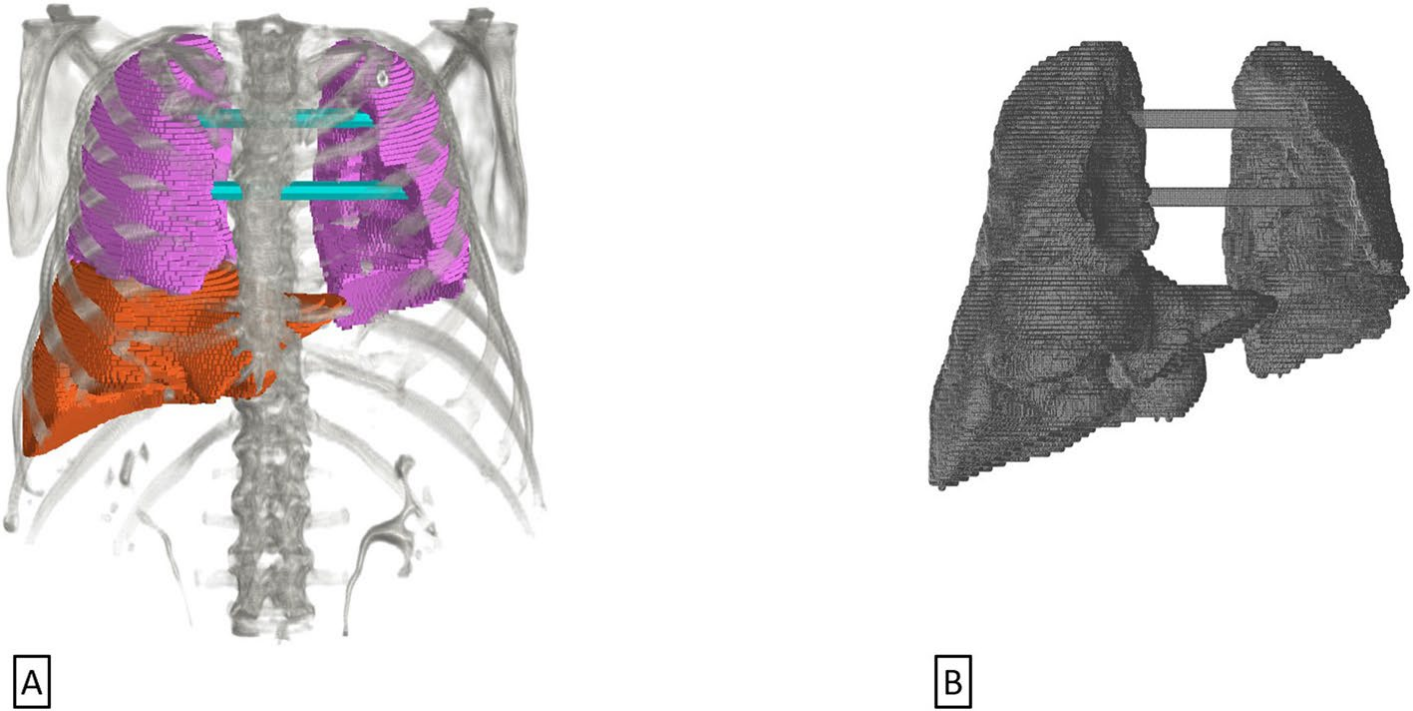
**A** TriDFusion (3DF)-rendered lung segmentation is depicted in pink, while the liver segmentation is shown in orange. The cyan reinforcement bars have been added using the TriDFusion (3DF) mask tool. **B** The segmentation results were exported as a 3D rendering in.stl format

In order to validate the 3DF LSF methodology and determine its accuracy, we constructed a phantom with fillable liver and lung compartments to serve as a “gold standard.” The phantom was fabricated using a 3D printer. TriDFusion (3DF) was used to segment the liver and lungs from the CT of a human subject and the resulting delineated surfaces were used to generate an .stl stereolithography model, as illustrated in Fig. 1. This conversion ensures compatibility with subsequent processing steps and facilitates seamless integration into the proper 3D printing workflows. The details of the liver and lungs CT phantom volumes are as follows: Liver volume is 1785 cm^3^ and lung volume is 2191 cm^3^ (left lung volume: 883 cm^3^ and right lung volume: 1308 cm^3^).

**Fig. 2:**
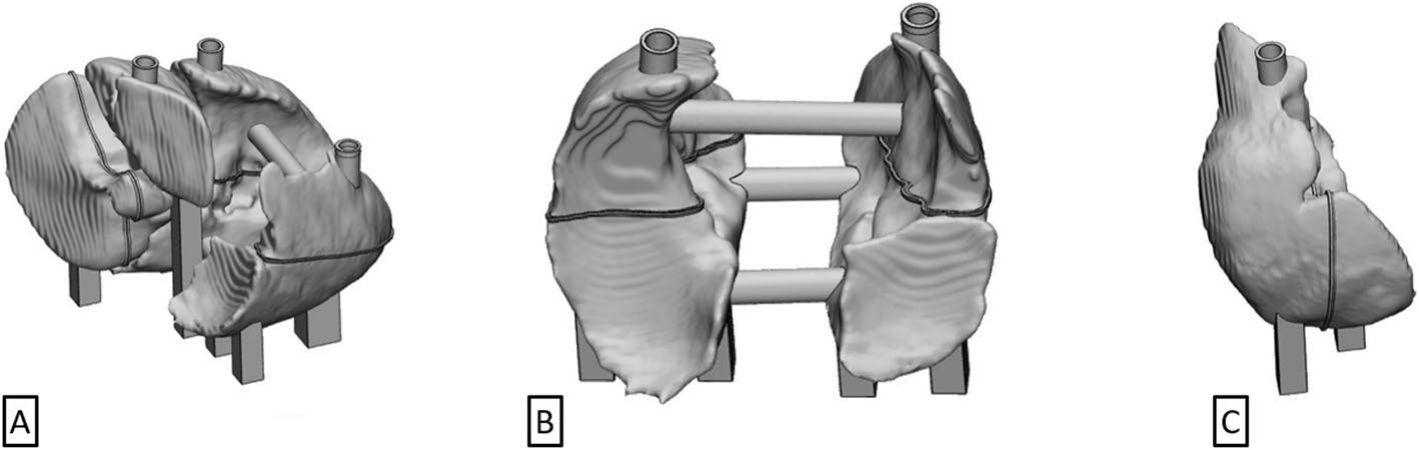
Medical physics engineering design of the lungs and liver, derived from the.stl model. **A** 3D volume rendering of the lungs and liver. **B** 3D volume rendering of the lungs. **C** 3D volume rendering of the liver

Utilizing the .stl mesh grid generated by TriDFusion (3DF), a detailed 3D model of the liver and lungs is fabricated using additive manufacturing techniques (Stratasys 3D printer model J850), as shown in Fig. 2. This process is conducted under the supervision of the Medical Physics Biomed Engineering group to ensure fidelity to the original anatomical structures.

**Fig. 3:**
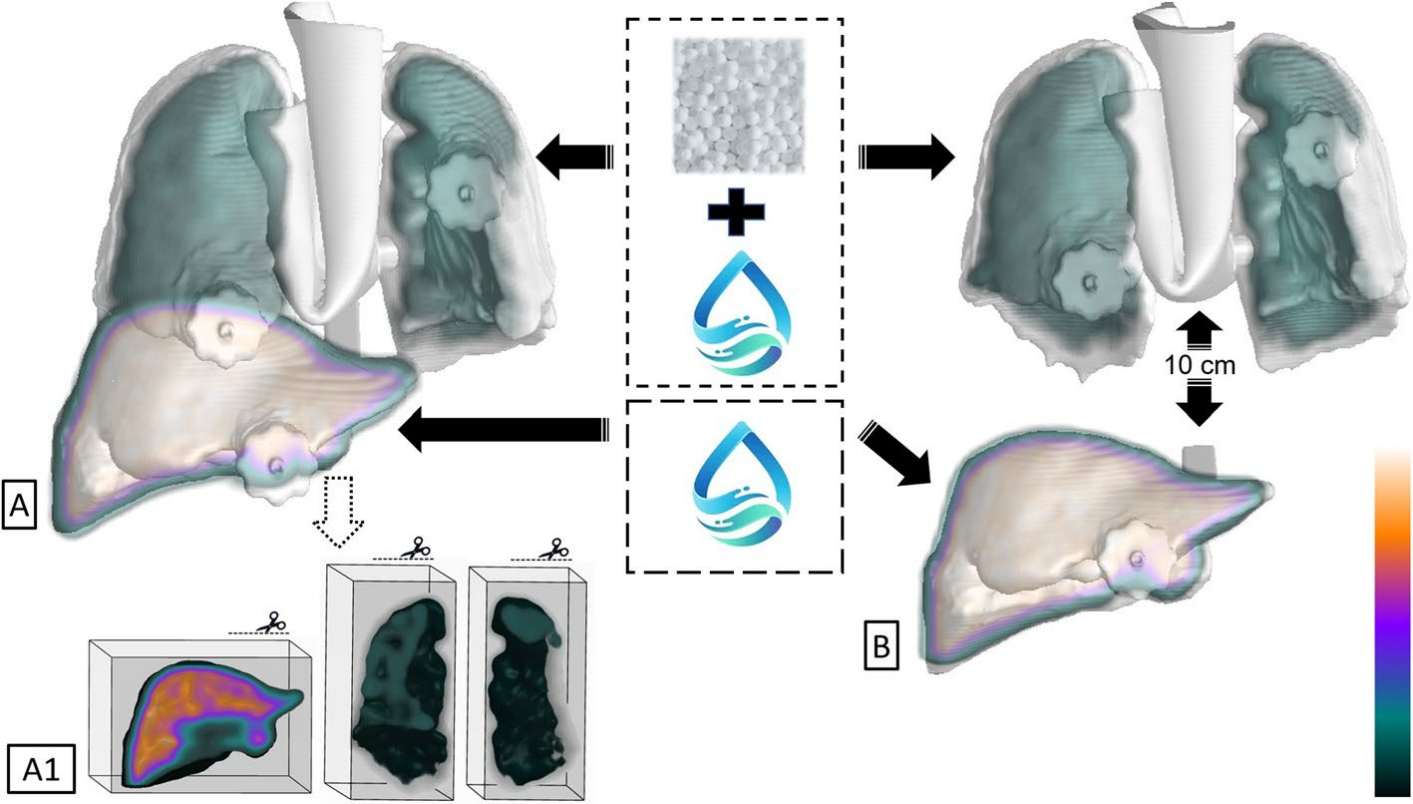
**A** Close proximity between the liver and lungs, accompanied by material insertion between the lungs. This is shown as a 3D isosurface of the computed tomography (CT) fused with a 3D maximum-intensity projection (MIP) of the single-photon emission computed tomography (SPECT) scan. **A1** A clipping plane window has been added to the image, showing a 3D volume rendering of the SPECT activity within the lungs and liver. **B** With increased distance between the lungs and liver

The liver of the fabricated 3D printed phantom is subsequently filled with water to simulate unit density, while the lung is filled with water and Styrofoam beads to simulate the density of lung tissue (Fig. 3A and B). This introduces varying scatter and attenuation properties that more closely resemble those found in the human body. A known activity of 99mTc [13] is then added to the lung and liver compartments to simulate a realistic LSF patient case. This phantom is then scanned on a SIEMENS Intevo SPECT/CT system using standard clinical acquisition protocols. The planar studies were acquired on a 512 × 512 matrix defaulted to either 5 min or 1,000,000 counts. The SPECT scans were acquired on a 128 × 128 matrix with 32 stops / 64 projections, 20 sec per stop.

### Four acquisition scenarios were performed

A Lungs filled with water and Styrofoam beads, and liver filled with water. A unit-density packing material (Superflab) is introduced between the lungs to serve as a scatter and attenuating medium. The spatial configuration of the lungs and liver are as in a patient.
B Same as scenario A, but with a 10 cm increased separation between the liver and lungs to eliminate overlap.
C Same as scenario A, but with liver and lungs both filled with water.
D Same as scenario C, but with a 10 cm increased separation between the liver and lungs.

### Acquisition A & B: lungs filled with water and Styrofoam beads, liver filled with water phantom

The HERMES Hybrid Recon 3.0.0 [14] software was employed for the reconstruction of the SPECT tomographic images. The Ordered Subset Expectation Maximization (OSEM) reconstruction used 5 iterations and 16 subsets. To investigate the importance of the different correction factors, the following reconstructions were performed:

1. With attenuation correction (AC), resolution recovery (RR), and scatter correction (SC), the standard reconstruction used for clinical studies.
2. With RR and SC, but no AC.
3. Without AC, RR, and SC, where no corrections are applied.

The LSF for the phantom-determined ratio measurements (the ground truth) is computed using the following equation:

$$LSF\% = \frac{{\left( {RLA \, - rlr} \right) \, + \, \left( {LLA \, - llr} \right)}}{{\left( {\left( {RLA - rlr} \right) + \left( {LLA - llr} \right) + \left( {LA - lr} \right)} \right)}} \times 100$$

where: RLA = Right lung activity, LLA= Left lung activity, LA = Liver activity, rlr= Right lung residual, llr= Left lung residual, lr= Liver residual

The LSF for the 3D SPECT-reconstructed image data is computed using the following equation:

$$LSF\% = \frac{{\left( {{\text{Total}}\;{\text{Lung}}\;{\text{Count}}} \right)}}{{({\text{Total}}\;{\text{Lung}}\;{\text{Count + Total}}\;{\text{Liver}}\;{\text{Count}})}} \times 100$$

The LSF for the 2D planar image data is computed using the following equation:

$$LSF\% = \frac{{\left( {{\text{Geo}}\;{\text{mean}}\;{\text{Lung}}\,{\text{Count}}} \right)}}{{{\text{(Geo}}\;{\text{mean}}\;{\text{Lung}}\;{\text{Count + Geo}}\;{\text{mean}}\;{\text{Liver}}\;{\text{Count)}}}} \times 100$$

where Geomean represents the geometric mean of both anterior and posterior planar counts.

## Results

### Acquisition A & B, with the lungs filled with water and Styrofoam beads

A summary of the measured results for the acquisitions performed is provided in Tables 1, 2, 3, 4. Tables 1 and 2 contain the results for the SPECT and planar acquisitions for the lung and liver patient configuration and Tables 3 and 4 are for the separate configuration. The SPECT results tables contain three rows representing: All three corrections, i.e., attenuation correction (AC), resolution recovery (RR), and scatter correction (SC); RR and SC without attenuation correction; and, finally, with no corrections applied.

**Table 1:** Acquisition A, 3D SPECT lung shunt fraction (LSF) accuracy

Image correction	Total lung count	Total liver count	LSF (%)	Accuracy (%)
1: AC, RR, SC	1,793,391.25	13,326,405	11.86	− 12.07
2: RR, SC	1,118,380.88	6,037,693	15.63	+ 15.88
3: No correction	1,060,934.88	5,738,963	15.60	+ 15.65

**Table 2:** Acquisition A, 2D planar lung shunt fraction (LSF) accuracy

Geomean lung count	Geomean liver count	LSF	Accuracy
212,709.6	712,039.1	23.00%	+ 70.51%

**Table 3:** Acquisition B, 3D SPECT lung shunt fraction (LSF) accuracy

Image correction	Total lung count	Total liver count	LSF (%)	Accuracy (%)
1: AC, RR, SC	1,611,104.5	11,983,855	11.85	− 12.15
2: RR, SC	1,001,383.5	5,662,139	15.03	+ 11.43
3: No correction	919,542.19	5,407,202	14.53	+ 7.72

**Table 4:** Acquisition B, 2D planar lung shunt fraction (LSF) accuracy

Geomean lung count	Geomean liver count	LSF (%)	Accuracy
327,558.4	875,329.1	27.2	+ 101.65

The data shows a significant change in the reconstructed total number of counts following application of the three corrections. Most of the change is a consequence of AC, as can be seen from the count differential between the AC, SC, and RR (row 1) versus SC and RR (row 2) and no correction (row 3) in Tables 1 and 3. The LSF, which is a ratio, is robust to the various reconstruction corrections applied. The largest deviation from the known LSF is +15.88% (Table 1) for the case of no AC is applied.

However, when the LSF was determined from 2D planar acquisition data, the deviations from the actual known LSF varied from +70.51% (Table 2) to +101.65% (Table 4).

The LSF based on the known syringe activities introduced into the two lung and liver phantom compartments (ground truth) is 13.5%. The measurement accuracy resulting from TriDFusion (3DF) analysis of the reconstructed SPECT-CT for the different reconstruction assumptions is presented in Tables 1 and 3, with the results for the planar scan analysis in Tables 2 and 4. Note that the SPECT acquisition with all corrections applied underestimates the LSF, whereas when no AC is applied, there is a significant overestimate of the LSF. Surprisingly, there was little difference between the LSF estimations between the close lung-liver configuration versus when the phantom components were separated, so as to eliminate scatter/attenuation cross talk.

Although we simulated the actual density and heterogeneity of human tissues with Styrofoam beads, water cannot penetrate the beads, and air pockets may still exist between them despite our precautions. We also acquired the phantom with the two lungs filled with water, without Styrofoam beads. The results of these measurements are presented in Table 5 in an addendum. The LSF accuracy for the AC reconstruction changed from  − 12.07% (Table 1) to  − 3.47% (Table 5 Addendum). And for the planar images, the accuracy improved from  + 70.51% (Table 2) to  + 21.81% (Table 6 Addendum). This suggests the importance of attenuation for accurate determination of the LSF.

### Comparison of 2D planar and 3D SPECT patient data

**Fig. 4:**
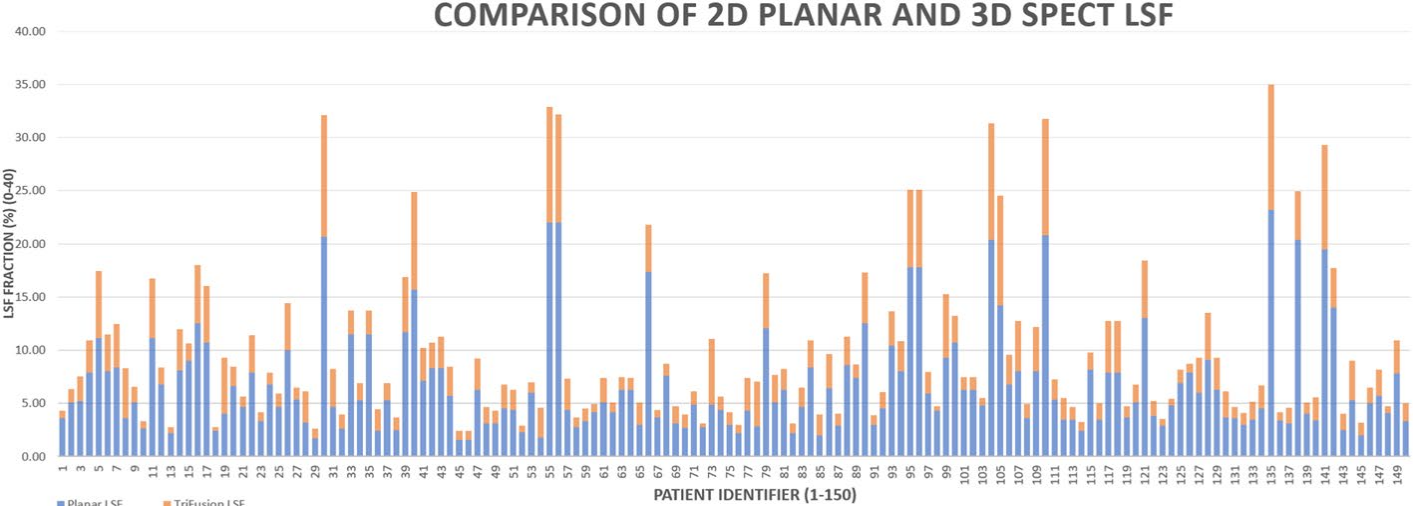
Comparison chart: 2D planar lung shunt fraction percentage versus 3D SPECT LSF percentage. The X-axis represents the case number, while the Y-axis displays the LSF as a percentage. The average LSF for the 2D planar is 6.85, while for the 3D SPECT, it is 2.92. The average percentage difference is 57%

We determined the LSF calculated from planar and SPECT-CT imaging using our standard clinical protocols for 150 patients who underwent 99mTc-MAA scans prior to 90Y-radioembolization. The results of this study are shown graphically in Fig. 4 for all patients estimated. The LSF is systematically higher when determined from ROI analysis from planar scans (blue line) compared to the TriDFusion (3DF) LSF (orange) from the SPECT-CT acquisitions. The percentage difference between the two is 57% and was calculated using:

$$PD = \frac{PL - TF}{{TF}} \times {1}00$$

where: *PD* = Percentage Difference, *PL* = Planar LSF, *TF* = TriDFusion LSF

We also calculated the Interquartile Range (IQR) to identify the spread of the middle 50% of the obtained data using the following formula:

- For the first quartile (Q1):
  $$Q{1} = \frac{{(n + {1})}}{{4}}$$
- For the third quartile (Q3):
  $${\text{Q3 = }}\frac{{{3}(n + {1})}}{4}$$

The Interquartile Range (IQR) is calculated using the formula:

$$IQR = Q{3} - Q{1}$$

where *Q*1 is the first quartile and *Q*3 is the third quartile.

Planar LSF:

- 1st Quartile (Q1): 3.50
- 3rd Quartile (Q3): 8.00
- Interquartile Range (IQR): 4.50

TriDFusion LSF:

- 1st Quartile (Q1): 1.16
- 3rd Quartile (Q3): 3.51
- Interquartile Range (IQR): 2.35

## Discussion

There have been a number of studies that have discussed the topic of LSF estimation. Torkian et al. [3] discusses some of the different methods to measure LSF, underscoring the fact that 2D Planar measurement is the most utilized because it is the easiest to perform. One consequence of planar LSF overestimation has been unnecessary dose reductions and even cancelation of 90Y radioembolization when the expected lung doses are too high, leading these authors to conclude that LSF calculations use 3D SPECT-CT whenever possible. Georgiou et al. [7] performed a detailed analysis of LSF calculations on 85 patients based on SPECT/CT versus 2D planar imaging showing differences similar to those found in our study.

Chaichana et al. [4] present one of the first CNN-based algorithm to automatically segment lungs, liver, and tumor from 99m Tc-MAA SPECT/CT images and a similar approach is adopted by Stella et al. [5] based upon holmium-166 images, an alternative radioembolization agent to the 99mTc-MAA/90Y-microsphere pair. Luu et al. [6] developed a nearly automatic CNN-based LSF quantification method for liver cancer SIRT treatment planning. The method trained with 60 liver cancer patients resulted in a reported LSF error relative to manual segmentation of 0.14%.

In our study cohort, we identified a significant systematic disparity between the LSF estimated from planar images of patients compared to estimates made from fully corrected 3D SPECT-CT data. Using a 3D-printed anthropomorphic liver-lung phantom, we demonstrated the accuracy of the LSF derived from 3D SPECT analysis and the inaccuracy of the LSF determined from 2D planar scans. It is common to perform 2D planar LSF estimates because of the ease and simplicity of the methodology. Conversely, the analysis of 3D SPECT-CT data is tedious, requiring more careful labor-intensive VOI analysis.

In this paper, we introduce a new computer algorithm to obtain the LSF accurately and rapidly from SPECT-CT acquisitions of the liver and lungs. The implementation of a new LSF machine learning tool within TriDFusion (3DF) presents a transformative solution by providing automated and comprehensive reports that facilitate fine-tuning of settings and quality control fusion features between SPECT and CT modalities. This advancement not only streamlines clinical workflows but also enhances the accuracy and consistency of the LSF analyses. The interactive report interface empowers users to customize analysis parameters and perform recalibrations, thereby fostering flexibility and enriching the clinical decision-making process. This underscores the importance of adapting practices to incorporate advancements in technology and methodology for more precise and effective patient care.

Conversely, tomographic SPECT acquisition LSF fraction is significantly more accurate than planar images. Even under optimal conditions, planar image LSF remains inaccurate. Given this significant disparity (57%), it may be prudent to reconsider the currently proposed threshold for percentage of administered activity reaching the lungs; our study suggests that higher activities of SIR could be administered without exceeding dose limits to normal lung parenchyma, which may translate to better response rates in liver parenchyma.

## Conclusions

TriDFusion (3DF) AI 3D Lung Shunt is an innovative computational imaging approach that seamlessly integrates the TriDFusion (3DF) Image Viewer and TotalSegmentor machine learning segmentation to accurately identify and analyze lung shunt. The utilization of AI-driven segmentation and breathing motion compensation masks enhances automation and efficiency, resulting in improved reproducibility and reduced analysis time.

TriDFusion (3DF) AI 3D Lung Shunt represents an advancement in computational imaging, paving the way for a more streamlined treatment planning workflow that will, by increasing LSF accuracy, lead to improvements in patient care. With its automated features, user-friendly interface, and robust quality control mechanisms, TriDFusion (3DF) holds great promise for improving lung shunt fraction analysis and advancing clinical practice in the fields of interventional radiology and nuclear medicine.

## Data Availability

TriDFusion (3DF) source code is available for download from GitHub(https://github.com/DICOMtools/TriDFusion.git). A MATLAB compiled version is also available for download (https://daniellafontaine.com/download/tridfusion/).

## Appendix

Acquisitions C and D, where the ratio of the absolute 99mTc activities added to the lung and liver yields an LSF value of 12%.

**Table 5:** Acquisition C, 3D SPECT lung shunt fraction (LSF) accuracy

Image correction	Total lung count	Total liver count	LSF (%)	Accuracy (%)
1: AC, RR, SC	1,771,384.62	13,536,898	11.57	− 3.47
2: RR, SC	830,743.5	6,055,443.5	12.06	+ 0.61
3: No correction	795,620.12	5,769,438.5	12.12	+ 1.11

**Table 6:** Acquisition C, 2D planar lung shunt fraction (LSF) accuracy

Geomean lung count	Geomean liver count	LSF (%)	Accuracy (%)
122,131.3	736,869	14.60	+ 21.81

## Declarations

### Copyright and collaboration

TriDFusion (3DF) is free, open-source software that has been developed and led by Daniel Lafontaine at Memorial Sloan Kettering Cancer Center. The software may be redistributed and/or modified under the terms of the GNU General Public License, as published by the Free Software Foundation, either version 3 of the License, or (at your discretion) any later version. It is for “educational,” “research,” and “not-for-profit” purposes only, which means any noncommercial and nonclinical use. Any contributed computer code may retain the copyright to TriDFusion (3DF) and users are responsible for their use of the code.

## References

[1] Sundram FX, Buscombe JR. Selective internal radiation therapy for liver tumours. Clin Med. 2017;17:449. 10.7861/clinmedicine.17-5-449 PMC6301920 28974597

[2] Tang X, Jafargholi Rangraz E, Coudyzer W, Bertels J, Robben D, Schramm G, Deckers W, Maleux G, Baete K, Verslype C, Gooding MJ. Whole liver segmentation based on deep learning and manual adjustment for clinical use in SIRT. Eur J Nucl Med Mol Imaging. 2020;47:2742–52. 32314026 10.1007/s00259-020-04800-3

[3] Torkian P, Ragulojan R, Woodhead GJ, D’souza D, Flanagan S, Golzaria J, Young S. Lung shunt fraction quantification methods in radioembolization: what you need to know. Br J Radiol. 2022;95:20220470. 35848755 10.1259/bjr.20220470 PMC9793490

[4] Chaichana A, Frey EC, Teyateeti A, Rhoongsittichai K, Tocharoenchai C, Pusuwan P, Jangpatarapongsa K. Automated segmentation of lung, liver, and liver tumors from Tc-99m MAA SPECT/CT images for Y-90 radioembolization using convolutional neural networks. Med Phys. 2021;48:7877–90. 34657293 10.1002/mp.15303 PMC9298038

[5] Stella M, van Rooij R, Lam MG, de Jong HW, Braat AJ. Automatic healthy liver segmentation for holmium-166 radioembolization dosimetry. EJNMMI Res. 2023;13:68. 37453996 10.1186/s13550-023-00996-1 PMC10349793

[6] Luu MH, Mai HS, Pham XL, Le QA, Le QK, Van Walsum T, Le NH, Franklin D, Le VH, Moelker A, Chu DT. Quantification of liver-lung shunt fraction on 3D SPECT/CT images for selective internal radiation therapy of liver cancer using CNN-based segmentations and non-rigid registration. Comput Methods Progr Biomed. 2023;233:107453. 10.1016/j.cmpb.2023.107453 36921463

[7] Georgiou MF, Kuker RA, Studenski MT, Ahlman PP, Witte M, Portelance L. Lung shunt fraction calculation using 99m Tc-MAA SPECT/CT imaging for 90 Y microsphere selective internal radiation therapy of liver tumors. EJNMMI Res. 2021;11:1–10. 34585259 10.1186/s13550-021-00837-z PMC8479035

[8] Riveira-Martin M, Akhavanallaf A, Mansouri Z, Bianchetto Wolf N, Salimi Y, Ricoeur A, Mainta I, Garibotto V, López Medina A, Zaidi H. Predictive value of 99mTc-MAA-based dosimetry in personalized 90Y-SIRT planning for liver malignancies. EJNMMI Res. 2023;13:63. 37395912 10.1186/s13550-023-01011-3 PMC10317941

[9] Lafontaine D, Schmidtlein CR, Kirov A, Reddy RP, Krebs S, Schöder H, Humm JL. TriDFusion (3DF) image viewer. EJNMMI Phys. 2022;9:72. 36258098 10.1186/s40658-022-00501-y PMC9579267

[10] Wasserthal J, Breit HC, Meyer MT, Pradella M, Hinck D, Sauter AW, Heye T, Boll DT, Cyriac J, Yang S, Bach M. Totalsegmentator: robust segmentation of 104 anatomic structures in CT images. Radiol: Artif Intell. 2023;5:e230024. 37795137 10.1148/ryai.230024 PMC10546353

[11] Isensee F, Jaeger PF, Kohl SA, Petersen J, Maier-Hein KH. nnU-Net: a self-configuring method for deep learning-based biomedical image segmentation. Nat Methods. 2021;18:203–11. 33288961 10.1038/s41592-020-01008-z

[12] Hertanto A, Zhang Q, Hu Y-C, Dzyubak O, Rimner A, Mageras GS. Reduction of irregular breathing artifacts in respiration-correlated CT images using a respiratory motion model. Med Phys. 2012;39:3070–9. 22755692 10.1118/1.4711802 PMC3360690

[13] Gandhi SJ, Babu S, Subramanyam P, Sundaram PS. Tc-99m macro aggregated albumin scintigraphy–Indications other than pulmonary embolism: a pictorial essay. Indian J Nucl Med. 2013;28:152–62. 24250023 10.4103/0972-3919.119546 PMC3822414

[14] Kangasmaa TS, Constable C, Hippeläinen E, Sohlberg AO. Multicenter evaluation of single-photon emission computed tomography quantification with third-party reconstruction software. Nucl Med Commun. 2016;37:983–7. 27128824 10.1097/MNM.0000000000000538

